# Protocol for a systematic review and meta-analysis of the prevalence of viral infections in acute apical abscesses

**DOI:** 10.1038/s41405-025-00320-0

**Published:** 2025-04-08

**Authors:** José Ángel Hernández-Mariano, Gustavo Adolfo Sánchez-Ramírez, Guillermo Cano-Verdugo, Myriam Angélica De la Garza-Ramos, Martín Andrés Chávez- Méndez, Claudio Peña-Soto, Mónica Alethia Cureño-Díaz

**Affiliations:** 1 Hospital Juárez de México, División de Investigación. Av. Instituto Politécnico Nacional No. 5160, Col. Magdalena de las Salinas, Del. Gustavo A. Madero, C.P. 07760, Ciudad de México, CDMX, México; 2 Universidad Autónoma de Nuevo León, Facultad de Odontología. Calle Dr. Eduardo Aguirre Pequeño y Silao S/N, Col. Mitras Centro, CP. 64460, Monterrey, Nuevo León México; 3 Universidad Científica del Sur, Facultad de Ciencias de la Vida y Salud, Carrera de Estomatología. Ctra. Panamericana Sur Km. 19, Villa El Salvador, CP. 15067, Lima, Perú; 4 Hospital Juárez de México, Dirección de Enseñanza e Investigación. Av. Instituto Politécnico Nacional No. 5160, Col. Magdalena de las Salinas, Del. Gustavo A. Madero, C.P. 07760, Ciudad de México, CDMX, Mexico

**Keywords:** Oral pathology, Dental epidemiology

## Abstract

**Background:**

There is no current consensus on the presence of viral infections in acute apical abscesses; therefore, this protocol for a systematic review and meta-analysis is designed to detail the procedures required to investigate the prevalence of viral infections in acute apical abscesses, a common dental condition characterized by pus accumulation due to bacterial infection. Viral infections in oral tissues have been linked to systemic health risks, including chronic inflammation and oncogenesis, which further emphasize the importance of understanding their role in acute apical abscesses.

**Methods/design:**

We adopted a systematic review and meta-analysis protocol design followed by PRISMA guidelines. A priori protocol was registered in PROSPERO with registry number: CRD42023468287. Inclusion criteria will be established according to the PICO framework; hence, we will include original articles with no restriction on publication date or population group. The selective screening of information will be conducted by peers, starting with titles, abstracts, and keywords, and finally reviewing the full text. The risk of bias will be assessed using the ROBINS tool, and the certainty of the evidence will be evaluated following the GRADE guidelines. We will perform a random-effects meta-analysis, utilizing the Freeman-Tukey double arcsine transformation, to estimate the pooled prevalence of viral infections in acute apical abscesses, assess heterogeneity using the *Q*-test and I² statistic, evaluate potential publication bias with funnel plots and Egger’s test, and conduct sensitivity analyses to ensure robust results.

**Discussion:**

At present, no consensus exists regarding the prevalence of viral infections in acute apical abscesses that could inform clinical dental practice. Moreover, the existing body of knowledge on this subject is notably limited. This approach is intended to provide data that will facilitate the improvement of clinical practice and serve as a methodological framework for studying various pathologies. By elucidating the prevalence of viral infections, the findings of this study could enhance diagnostic accuracy and inform more targeted and effective treatment strategies, ultimately improving patient outcomes.

## Introduction

The oral cavity is known to be the second most diverse microbial environment, encompassing approximately 700 species, including bacteria, fungi, viruses, and protozoa. This environment is notably intricate, with microorganisms colonizing tooth surfaces, soft oral mucosal tissues, and, as recent studies have emphasized, dental abscesses. These microbes play a vital role in both oral and systemic health [1–3].

Acute apical abscesses are a common issue in both dentistry and oral medicine, marked by the accumulation of pus at the apex of a tooth due to bacterial infection of the pulp tissue [4]. While these abscesses are extensively studied, there is growing interest in the role of viruses within them. Viruses, often neglected in discussions about dental infections, could significantly affect the development and severity of acute apical abscesses. They may act as reservoirs for viral infections that could go unnoticed by patients and healthcare providers alike [5, 6].

Research has identified the presence of various viruses, including Human Papillomavirus (HPV), Epstein-Barr Virus (EBV), and Cytomegalovirus (CMV), in potentially malignant lesions of the oral cavity and periodontium [7–9], as well as Human Herpesvirus (HHV) in Hodgkin’s lymphoma [10]. However, the prevalence of these viruses in acute apical abscesses—a frequent problem in dental settings—varies. Gaining a comprehensive understanding of the different viruses present in acute apical abscesses is essential to mitigate potential risks, as some of these viruses are associated with a high carcinogenic potential in the oral cavity.

Therefore, this manuscript aims to outline the steps necessary to conduct a systematic review and meta-analysis. This approach is intended to provide data that will facilitate the improvement of clinical practice and serve as a methodological framework for studying various pathologies.

## Methods study design

We adopted a systematic review and meta-analysis design followed by the Preferred Reporting Items for Systematic Reviews and Meta-Analyses (PRISMA) guidelines [11]. The study protocol was registered on the International Prospective Register of Systematic Reviews (registry number: CRD42023468287).

### Study selection

We will conduct electronic searches across PubMed, Google Scholar, Cochrane Library, and Elsevier databases to identify studies focused on the presence of viral infections in acute apical abscesses. The search strategy is detailed in Table 1.

**Table 1:** Search strategy.

Database	Search strategy
PubMed	(“abscess”[MeSH Terms] OR “abscess”[All Fields] OR “abscesses”[All Fields] OR “abscessation”[All Fields] OR “abscessed”[All Fields] OR “abscessing”[All Fields]) AND “hpv”[All Fields]
Google Academic	“abscess” AND “apical” AND “HPV”
Cochrane library	HPV abscess
Elsevier	(abscess) AND (HPV)

Relevant publications will be searched from the inception of the databases up to June, 2025, with no restrictions on date or language. The titles and abstracts of the retrieved documents will be independently screened by two authors (G.A.S.R. and M.A.C.D.) to evaluate their relevance to our research objective. Following this, the authors will review the full text of all potentially relevant studies, applying the eligibility criteria to select studies for qualitative data synthesis. Any discrepancies between the authors reviewing the full texts will be resolved through consensus between C.P.S. and M.A.C.M.

### Eligibility criteria

The eligibility criteria for the studies will be developed based on the components of the PECOS statement (population, exposure, comparators, outcome, and study design) [12], addressing the research question: “What is the prevalence of viruses in acute apical abscesses in humans?” (Table 2). We will include all original studies that investigate the presence of any type of virus in acute apical abscesses. Reviews, conference papers, and comments will be excluded.

**Table 2:** Eligibility criteria and data ítems.

PECO element	Inclusion and exclusión criteria	Data assumption
P Participants/population	Inclusion: Patients with acute apical abscesses Exclusion: Patients with periodontal abscesses, gum, or any other region of the oral mucosa	They were defined as patients who attended a dental consultation and exhibited the presence of at least one acute apical abscess.
E Exposure	Inclusion: Performing PCR on acute apical abscesses for viral gene determination Exclusion: Failure to perform viral gene identification	It was defined as the implementation of molecular testing through PCR in the patient to identify the type of virus present.
C Comparator/control	Inclusion: Healthy patients without the presence of acute apical abscesses. Exclusion: Does not apply.	They were defined as users who did not attend dental consultation, did not have the presence of any acute apical abscesses, and did not undergo molecular PCR analysis at the same time.
O Outcome	Inclusion: Prevalence of viruses in acute apical abscesses. Exclusion: Does not apply.	It was defined as the number of confirmed cases of acute apical abscesses with the type and subtype of virus present.
T Time	Inclusion: No restriction on the age of the data. Exclusion: Does not apply.	It is the publication time considered for data search.
S Study	Inclusion: only original articles Exclusion: review articles	It was defined as the types of manuscripts included that conducted experiments or observations.

Periodontal abscesses will also be excluded to maintain a focused scope, as their etiology and clinical management differ significantly from acute apical abscesses. The emphasis on original studies ensures the inclusion of primary data, minimizing

biases associated with secondary analyses and providing a robust foundation for systematic review and meta-analysis.

**Fig. 1:**
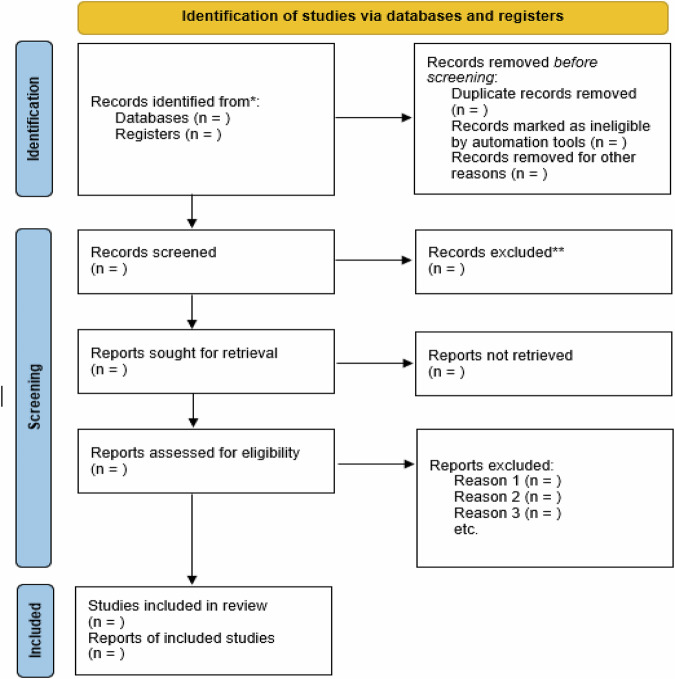
Flow diagram to be used to describe the process of searching and selecting scientific articles.

The process of searching and selecting studies to be included in this review will be summarized in a flow diagram based on the Preferred Reporting Items for Systematic Reviews and Meta-Analyses (PRISMA) guidelines (see Fig. 1).

### Data extraction and quality assessment

Data from the eligible studies will be extracted independently by two authors (M.A.G.R. and J.A.H.M.) using a pre-piloted standard form. Information to be extracted will include: first author, year of publication, country where the study was conducted; sample size and sex of participants; age of participants; prevalence outcomes with virus subtypes (if reported); conclusions, findings, participants’ HIV status, and physical status according to the ASA Classification System, oral hygiene status, and risky sexual practices. When data of interest is not reported, authors will be contacted via email.

To ensure inter-rater reliability during the data extraction process, two authors (M.A.G.R. and J.A.H.M.) will extract data independently using a standardized form. Discrepancies between the extracted data will be resolved through discussion and, if necessary, consultation with a third author (G.C.V.). Cohen’s kappa statistics will be calculated to quantify the level of agreement between the reviewers, with a kappa value above 0.75 considered indicative of excellent agreement.

Risk of bias in each study will be evaluated using the Risk Of Bias In Non-randomized Studies—of Exposures (ROBINS-E) tool [13]. ROBINS-E tool assesses seven domains of bias: (1) confounding, (2) selection of participants into the study, (3) classification of exposures, (4) departures from intended exposures, (5) missing data, (6) measurement of outcomes, and (7) selection of the reported result.

The information from the included articles and the evaluation of their quality will be used to develop the qualitative synthesis of the data.

The certainty of the evidence will be assessed using the GRADE framework (Grading of Recommendations Assessment, Development, and Evaluation) [14], considering the study design, risk of bias, imprecision, inconsistency, indirectness, and publication bias to rate the level of evidence. The two aforementioned processes will be conducted by M.A.C.M. and supervised by G.C.V and M.A.C.D.

### Statistical analysis

If sufficient quantitative data are available, we will estimate the pooled prevalence of viral infections in acute apical absences, and its 95% confidence interval based on the data reported in the studies will be included in this review. The pooled prevalence will be estimated by random-effects meta-analysis to account for heterogeneity across studies. Due to the potential low frequency of some viral infections, we will use the Freeman-Tukey double arcsine transformation in the meta-analysis to stabilize the prevalence variations reported in each primary study, allowing for a robust estimate of the pooled prevalence [15]. As a secondary analysis, we will conduct independent meta-analyses to determine the pooled prevalence of viral infections in acute apical abscesses according to the type of virus.

The Freeman-Tukey double arcsine transformation was selected for its ability to stabilize variances when dealing with prevalence rates that are near 0% or 100%, which can otherwise lead to misleading results in meta-analyses. This approach ensures that the pooled prevalence estimates are robust and not disproportionately influenced by studies reporting extreme values.

Statistical heterogeneity among the selected studies will be assessed using the Q-test (with *p* > 0.10 indicating the presence of heterogeneity) and the I² statistic (range 0–100%) [16]. According to Cochrane’s criteria, an I² < 30% indicates low heterogeneity, an I² between 30 and 60% indicates moderate heterogeneity, and an I² ≥ 75% indicates considerable heterogeneity. We will perform subgroup meta-analyses and meta-regression analyses in the presence of moderate or greater heterogeneity to identify potential sources of heterogeneity, such as geographic region, ASA Physical Status Classification, patient age, underlying medical conditions, poor oral hygiene, weak immune system, and risky sexual practices. These subgroup analyses aim to reveal unique trends or risk factors associated with viral infections in acute apical abscesses, which may provide insights into specific patient populations or clinical scenarios that warrant tailored interventions.

Potential publication bias will be assessed through visual inspection of funnel plots to detect asymmetric patterns and will be complemented with Egger’s test (with a *p*-value < 0.10 indicating publication bias) [17]. As a sensitivity analysis, we will explore the effect of individual studies on the pooled prevalence of viral infections in acute apical abscesses by excluding one study at a time and re-estimating the pooled prevalence. All statistical analyses will be conducted using STATA software. This analysis will be conducted by J.A.H.M. and supervised by C.P.S.

## Conclusion

We anticipate identifying a significant prevalence of viral infections within acute apical abscesses, with variations in the prevalence rates depending on the specific viral subtypes involved. We expect the pooled prevalence estimates to reveal noteworthy heterogeneity across different geographic regions, patient demographics, and clinical conditions. The analysis may uncover correlations between viral presence and specific risk factors, including poor oral hygiene, immunocompromised status, and risky sexual behaviors. In addition, we predict that the systematic review and meta-analysis will underscore the necessity for incorporating viral diagnostics in the routine evaluation of acute apical abscesses, given the potential implications for both oral and systemic health. Ultimately, the findings are expected to contribute valuable insights into the role of viruses in dental infections, potentially influencing clinical practices and future research directions.

## Supplementary information

PRISMA Checklist

## Data Availability

The data generated when this systematic review and meta-analysis is completed will be available in an online repository (Mendeley Data).
