# Correction: Protocol for a systematic review and meta-analysis of the prevalence of viral infections in acute apical abscesses

**DOI:** 10.1038/s41405-025-00334-8

**Published:** 2025-04-22

**Authors:** José Ángel Hernández-Mariano, Gustavo Adolfo Sánchez-Ramírez, Guillermo Cano-Verdugo, Myriam Angélica De la Garza-Ramos, Martín Andrés Chávez- Méndez, Claudio Peña-Soto, Mónica Alethia Cureño-Díaz

**Affiliations:** 1 Hospital Juárez de México, División de Investigación. Av. Instituto Politécnico Nacional No. 5160, Col. Magdalena de las Salinas, Del. Gustavo A. Madero, C.P. 07760, Ciudad de México, CDMX, México; 2 Universidad Autónoma de Nuevo León, Facultad de Odontología. Calle Dr. Eduardo Aguirre Pequeño y Silao S/N, Col. Mitras Centro, CP. 64460, Monterrey, Nuevo León México; 3 Universidad Científica del Sur, Facultad de Ciencias de la Vida y Salud, Carrera de Estomatología. Ctra. Panamericana Sur Km. 19, Villa El Salvador, CP. 15067, Lima, Perú; 4 Hospital Juárez de México, Dirección de Enseñanza e Investigación. Av. Instituto Politécnico Nacional No. 5160, Col. Magdalena de las Salinas, Del. Gustavo A. Madero, C.P. 07760, Ciudad de México, CDMX, Mexico

**Keywords:** Oral pathology, Dental epidemiology

Correction to: *BDJ Open* 10.1038/s41405-025-00320-0, published online 08 April 2025

In this article the author’s name Claudio Peña-Soto was incorrectly written as Claudio Soto-Peña. The original article has been corrected.

